# Guiding pluripotent stem cell therapies past the immune system

**DOI:** 10.1093/stcltm/szag051

**Published:** 2026-08-05

**Authors:** Hannah A Pizzato, Deepta Bhattacharya

**Affiliations:** Center for Advanced Molecular and Immunological Therapies, University of Arizona, Tucson, AZ 85724, United States; Center for Advanced Molecular and Immunological Therapies, University of Arizona, Tucson, AZ 85724, United States; Department of Immunobiology, University of Arizona College of Medicine, Tucson, AZ 85724, United States; Department of Surgery, University of Arizona College of Medicine, Tucson, AZ 85724, United States; BIO5 Institute, University of Arizona, Tucson, AZ 85724, United States; University of Arizona Cancer Center, Tucson, AZ 85724, United States

**Keywords:** pluripotent stem cells, transplantation immunology, graft rejection, cell transplantation, regenerative medicine

## Abstract

Pluripotent stem cell (PSC)-based therapies hold the potential to unlock cures for numerous diseases, including, but not limited to, Parkinson’s disease, macular degeneration, heart failure, type 1 diabetes, and cancer. Yet as protocols to differentiate PSCs into therapeutically useful cell types have progressed rapidly, immunological rejection remains a major barrier that may limit the widespread use of such PSC-based therapies. In recent years, strategies to genetically modify PSCs to prevent immunological rejection of the downstream cell product have become a point of emphasis. Here, we provide an immunological perspective on these strategies, discussing the breadth of rejection mechanisms that have been uncovered through decades of research and the relative simplicity of designing PSC immune evasion strategies to circumvent these mechanisms. We focus in particular on how these strategies apply to the treatment of type 1 diabetes.

## Graphical Abstract

**Graphical Abstract:**
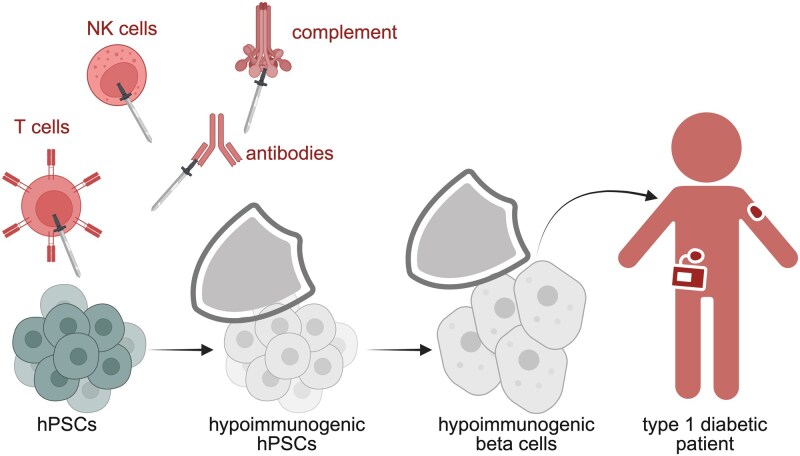

Significance statement Type 1 diabetes affects millions of people globally. Currently, beta cell transplants are not widely used due to limited availability of donor cells and risks associated with the lifelong immunosuppressive therapy required to prevent transplant rejection. As pluripotent stem cell-derived beta cells have started to address the problem of scalability, gene editing to protect cells from immunological rejection has stepped into the spotlight. Here, we define the role the immune system plays in transplant rejection, summarize current strategies to prevent immunological rejection, and address how these advances are starting to translate into clinical trials for hypoimmunogenic pluripotent stem cell-derived beta cells as a cure for type 1 diabetes.

## Introduction

To begin, it is helpful to consider the immune system in the context of an evolutionary arms race with infectious microbes. Some 400 million years ago, the first organisms began to develop adaptive immune systems, enabling highly tailored responses to pathogens and establishing immunological memory.^1^ Microbes responded with a variety of countermeasures, which in turn triggered evolutionary rebuttals by hosts. This still ongoing back and forth has shaped the immune system into an immensely complex network with backup after backup mechanism. As immunologists, the notion that a few genetic modifications would allow allogeneic cells to become invisible to the immune system and subvert millions of years of evolution initially seemed implausible. Thus, our own initial goals were to uncover new rejection mechanisms more than any realistic expectation of fully evading immunological rejection of pluripotent stem cell (PSC) therapies. Yet our views have become more optimistic as results from our group and others have been reported, particularly as they relate to the treatment of type 1 diabetes (T1D).

## Mechanisms of transplant rejection

Strategies to engineer PSCs and downstream beta cells to evade immunological rejection are instructed by decades of clinical and mouse transplantation studies. Broadly, there are three types of transplant rejection: hyperacute, acute, and chronic.^2^ Hyperacute rejection occurs in minutes to hours post-transplant and is characterized by pre-existing antigraft antibodies. Acute rejection takes weeks to a few months, while chronic happens over months and years. Both acute and chronic rejection can be antibody- and cell-mediated; however, antigraft antibodies are generated after transplantation.

The best appreciated immunological mechanism of transplant rejection is direct T cell-mediated recognition of mismatched human leukocyte antigens [HLA,^3^ or major histocompatibility complexes (MHC) in other species^4^] HLA molecules are highly polymorphic, expressed on the cell surface, and present peptides to T cells.^5^ Each unique HLA allele presents different peptide repertoires, creating diversity that is advantageous at the population level in that an escape mutation in any pathogen-derived peptide is unlikely to confer the same advantage in another individual. HLA molecules are further subdivided into HLA-I and -II. HLA-I is expressed on almost all nucleated cells and presents cytoplasmic peptides to cytotoxic CD8^+^ T cells. HLA-II is largely restricted to antigen-presenting cells (APCs) and presents vesicular peptides to helper CD4^+^ T cells. While the presentation of non-self-peptides promotes immune responses to pathogens and malignancies, it also causes allograft rejection, as a high frequency of the T cell repertoire recognizes antigens from HLA-mismatched donors.^6^ This rejection is likely an unintended byproduct of immune recognition mechanisms evolutionarily selected for antimicrobial responses. However, it is noteworthy that genetically nondiverse Tasmanian devil populations have been decimated by inter-individual cancer transmission,^7^^,^^8^ suggesting there may also be some selective advantage for species to have evolved allograft rejection mechanisms. T cell-mediated rejection caused by mismatched HLA is often acute and sometimes chronic. Additionally, due to polymorphisms across the population in genes encoding normal self-proteins, some peptides presented by a donor may differ in sequence from those presented by the recipient, even when HLAs match. These mismatched peptides are minor histocompatibility antigens and represent another opportunity for the immune system to recognize non-self.

HLA-ablation is almost certainly necessary to prevent T cell recognition of ‘universal donor PSCs yet is unlikely sufficient to prevent complete immunological rejection. Mouse MHC-deficient skin allografts are rejected almost as quickly as MHC-mismatched allografts.^9^^,^^10^ Therefore, direct MHC:T cell interactions are not the sole drivers of rejection. Again, we can gain clues on potential rejection mechanisms from evolution. Many viruses have evolved mechanisms to suppress HLA-I expression,^11^ which in turn triggered host adaptations. One elegant strategy employs natural killer (NK) cells, which are normally inhibited by HLA-I.^12^ NK cells express a panoply of inhibitory and activating receptors that regulate function. The ligands for these receptors provide targets for modifications to evade NK cell killing. Yet NK cells alone cannot explain how rapidly mouse skin allografts are rejected.^10^ Rather, CD4^+^ T cells were essential to mediate rejection, even though the grafts did not express HLA-II. These data suggest additional complex and indirect pathways to transplant rejection, further underscoring the difficulty of overcoming immune responses.

One potential mechanism by which CD4^+^ T cells indirectly cause rejection is by helping B cells generate antigraft antibodies. APCs can engulf cells from the graft, then present peptides on HLA-II to activate alloreactive CD4^+^ T cells. These T cells cooperate with B cells that have been partially activated by graft material to yield antigraft antibodies, leading to acute or chronic rejection. These graft-reactive antibodies can engage other immune pathways, including NK cells (through antibody-dependent cellular cytotoxicity), phagocytes (through Fc receptor-promoted engulfment), and complement.^13–15^ When complement is deposited on the surface of target cells, it can directly lyse the cell while also contributing to activating other immune cells. There are three pathways of the complement cascade; the antibody-mediated classical pathway is the most studied in organ transplants, but the antibody-independent alternative and lectin pathways also contribute to rejection.^16–19^ Additionally, more daunting mechanisms of rejection also exist. For example, donor-derived antigens can be indirectly captured by neighboring host endothelial cells and cross-presented to CD8^+^ T cells, leading to an immunological attack on blood vessels and graft loss from ischemia.^20^

These are just several known examples among the multitude of transplant rejection mechanisms, providing justification to our initial pessimism regarding generating ‘universal donor PSCs. Yet, the antecedent research on basic immunological recognition mechanisms, viral immune evasion strategies, and transplantation immunology provided guidance on starting points for pathways to target in PSCs, which could in turn reveal the next sets of backup rejection mechanisms.

## Current immune evasion strategies

**Figure 1.**
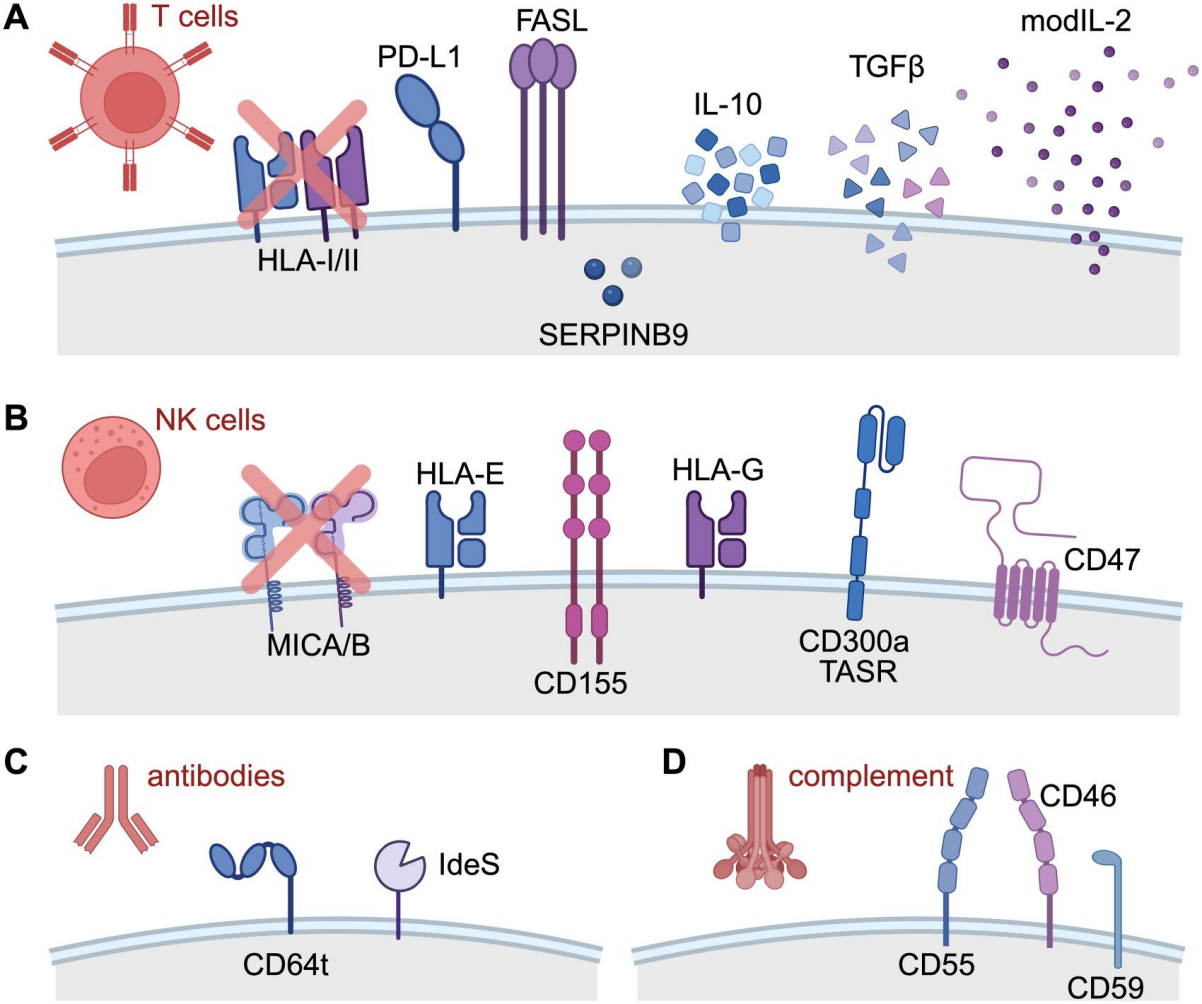
Inhibitory mechanisms to protect PSC-based therapies from T cell, NK cell, antibody, and complement-mediated rejection. Various strategies have been employed or proposed to prevent immunological rejection of PSC-based therapies. (A) Strategies to prevent T cell recognition and killing include gene editing to ablate HLA-I and -II; to express PD-L1, FASL, and/or SERPINB9; or to secrete IL-10, TGFβ, and modIL-2. (B) To evade NK cell rejection, MICA and MICB can be knocked out and/or HLA-E, CD155, HLA-G, CD300a TASR, and/or CD47 can be expressed. (C) CD64t or IdeS expression can inhibit antibody-mediated rejection. (D) CD55, CD46, and CD59 can provide protection from complement-mediated killing. Figures were created with BioRender.

With decades of work supporting HLA-matching to prevent T cell-mediated rejection, most strategies start by inhibiting T cells. Most PSC immune evasion strategies employ genetic ablation of HLA molecules directly^21^ or factors required for their surface expression^22–25^ (Figure 1A). β2-microglobulin (β2M) is a common subunit for HLA-I, and many studies have demonstrated its loss in PSCs is effective in preventing HLA-I expression.^22^^,^^25^^,^^26^ CIITA is a transcription factor required for HLA-II expression; thus, several groups have targeted this gene in PSC-editing strategies.^22^^,^^25^^,^^26^

Alternatively, several groups have employed strategies to induce T cell death, tolerance, and/or ignorance (Figure 1A). For instance, one study overexpressed programmed death-ligand 1 (PD-L1) on otherwise unedited human induced PSCs (iPSCs).^27^ Other factors such as Fas ligand (FASL) and serine proteinase inhibitor B9 (SERPINB9) have been employed to kill and inhibit T cells.^28^ In other approaches, edited cells express or secrete molecules that tolerize the immune response. For example, human embryonic stem cells (ESCs) were edited to secrete interleukin-10 (IL-10), transforming growth factor β (TGFβ), and modified IL-2 (modIL-2) to recruit regulatory T cells.^29^ Remarkably, diabetes was reversed upon differentiation of these cells into islets and transplantation into non-obese diabetic mice.^29^ Yet as described previously, there is little reason to believe evading direct T cell recognition will be sufficient to prevent rejection.

Therefore, most strategies ablating HLA-I also include methods to evade NK cells (Figure 1B), which can become activated by missing self. Expression of HLA-E, a non-polymorphic HLA-I molecule with a limited peptide repertoire, is a well-established strategy to engage the inhibitory NKG2A receptor.^25^ Other strategies include expression of alternate NK cell-inhibitory molecules, such as CD155^30^ or HLA-G.^25^ Conversely, other strategies have ablated well-studied ligands of the activating NKG2D receptor on NK cells, such as MICA and MICB.^25^ The challenge with each of these strategies is that they are independently unlikely to prevent all NK cell activity. For example, NKG2A is absent on ∼20%-80% of NK cells, leaving this subset unaffected by HLA-E.^31^ NKG2D is expressed by all NK cells, but its blockade reduces cytotoxicity by only ∼50%.^32^ This diversity across both the NK cell population within an individual and person-to-person variability across the population requires what seems to be a multipronged approach to evade NK cells. Yet encouragingly, engagement of a single molecule, CD300a, which is expressed by all human NK cells, was recently shown to prevent cytolytic activity.^33^ This would be an example of an approach to prevent NK cell-mediated rejection that was much simpler than anticipated. Perhaps because CD300a is normally engaged by specific host phospholipids,^34^^,^^35^ viruses have not managed to co-opt this inhibitory pathway.

CD47 overexpression has also been reported by one group as an alternate pan-NK cell inhibitory strategy.^36^ This strategy was reported as successful in a wide variety of experiments, including mouse organ transplants, engraftment of mouse and human PSC-derived cells/tissues into immunocompetent or humanized mice, and allogeneic transplants of chimeric antigen receptor (CAR)-T cells and pancreatic beta cells.^22–24^^,^^37^^,^^38^ Through this work, it was reported that CD47 inhibits NK cell killing by binding signal regulatory protein α (SIRPα), which can be induced by IL-2 *in vitro.*^36^

That endogenous CD47 expression would be limiting, and overexpression required to engage SIRPα on NK cells is puzzling. CD47 is nearly ubiquitously expressed at high levels by most cells,^39^ certainly sufficient to prevent phagocytosis by SIRPα^+^ phagocytes.^40^ Indeed, an independent study reported no NK cell inhibition by overexpressed CD47 in HLA-deficient T cells.^33^ Reciprocally, inhibiting CD47-SIRPα interactions only minimally enhanced the activity of IL-2-activated NK cells *in vitro* against beta cells.^29^ Others have tested CD47 overexpression in transplants with little success. In a single patient, a pig heart engineered to express human proteins, including CD47, was transplanted into a human with immunosuppression.^41^^,^^42^ When the graft was recovered after the patient died of heart failure 60d post-transplant, there were multiple signs of rejection, including severe complement deposition. HLA-I and -II-deficient hESCs overexpressing mouse CD47 were rejected within 11d of transplantation in immunocompetent mice.^26^ Similarly, we found that mouse CD47 overexpression on HLA-ablated hESCs provided no added protection over HLA-ablation in teratoma assays in immunocompetent mice.^25^ Moreover, when kidney transplants were performed in a rat model of ischemia reperfusion injury, CD47 blockade actually improved graft survival relative to controls.^43^ Importantly, none of these studies attempted an exact reproduction of the original studies. Nonetheless, that CD47 overexpression failed to protect HLA-deficient grafts against NK cell rejection in these independent studies raises concerns about the broad applicability of this strategy.

Avoiding direct recognition by T cells and NK cells is still not expected to fully protect grafts, as antibody responses can be initiated indirectly and lead to graft rejection through their effector functions. Strategies to limit antibody-mediated rejection (Figure 1C) are less common but, in our view, essential. Normally, antibodies bind to cellular targets (often foreign HLA) via their Fab arms and then recruit and engage effector functions with their Fc tails. In one approach, a truncated high-affinity Fc receptor (CD64t) was expressed to effectively turn antibodies in the wrong direction.^44^^,^^45^ By inverting antibodies, the goal is to prevent engaging Fc-mediated effector functions. Yet the concentration of IgG in human serum is quite high at several mg/mL. It is hard to see how CD64t on a graft would avoid becoming saturated quickly upon transplantation. Emerging enzymatic strategies, such as IdeS from *Streptococcus pyogenes*,^46^ may be a more promising strategy since they can processively remove Fc fragments from antibodies. Reciprocally, cell surface proteins such as CD55 can decay and prevent enzymatic activation of the antibody-dependent classical complement pathway^25^ (Figure 1D). Expression of other complement regulators, such as CD46 and CD59, can further limit the alternative pathway and membrane attack complex, respectively, to promote graft survival.^25^

Given the likely existence of still other undiscovered mechanisms of graft loss, we were expecting additional pathways to stymie our efforts. Yet we were pleasantly surprised to find the hPSCs we engineered to inhibit T cells, NK cells, phagocytes, and complement were not rejected by immunocompetent mice.^25^ Through this work, we found inhibiting phagocytes (via CD47, PD-L1, and CD24) was unnecessary for graft survival, but inhibition of T cells, NK cells, and complement was sufficient.^25^

Some technical challenges still remain with gene editing for immune evasion. These challenges include transgene silencing, off-target mutations, chromosomal rearrangement, and nonspecific integration, among others.^47^ Still, as more immune evasion strategies are reported, we continue to gain optimism that evading immunological rejection might not be as implausible as we initially thought. These strategies still require testing in clinical trials, but there are multiple distinct approaches that can be taken to evade T cells, NK cells, and antibodies/complement to allow graft survival.

## Safety considerations

Projecting forward, it is worth considering the potential safety risks of cells invisible to the immune system. Infections and oncogenic transformation of minimally immunogenic cells are often raised as safety concerns that may limit the utility of these approaches.^48^ We discuss these concerns from an immunologic perspective and frame them by considering the circumstances in which the patient would be worse off than if they had never received the transplant.

Should minimally immunogenic cells become infected, with the strategies commonly employed, pattern recognition receptors (PRRs) of the innate immune response remain intact. These PRRs allow the recognition of conserved molecular patterns associated with pathogens and host cell damage or death. Upon recognition of these motifs, inflammatory signals are produced to suppress pathogen replication and spread the antiviral state to neighboring cells.

As discussed above, priming of the adaptive immune response is usually initiated indirectly by APCs engulfing other cells, often after infections. Because indirect antigen presentation occurs irrespective of whether the infected cells express HLA, the impact of infected HLA-deficient grafts on systemic immunity is expected to be limited. Systemic pathogen-specific antibodies would be made, and T cells would patrol and limit infection in all cells in the body except the graft. Contrast this to when systemic immunosuppression is administered to prevent rejection. Restricting immune evasion only to the transplant seems preferable. It is possible that viral infection and cell-to-cell spread amongst transplanted cells could occur unchecked without direct CD8^+^ T cell recognition, leading to cell death and graft loss. While immunopathology and bystander damage to surrounding tissues is possible, this situation would likely represent a ‘reset to the status quo prior to transplantation and might not necessarily preclude a second transplant after clearance of the infection.

A more problematic scenario could arise with oncogenic viruses. These viruses often possess mechanisms to keep cells alive, and direct control by the immune system is essential to reduce the likelihood of oncogenesis. Transplantation of minimally immunogenic cells for which oncogenic viruses have tropism should, thus, be considered only with caution. For example, cells may need to be ablated for viral entry and/or fusion receptors. We are unaware of evidence that known oncogenic viruses, such as human papillomavirus, Epstein–Barr, human T lymphotropic, Merkel cell polyoma, or Kaposi’s sarcoma viruses, can productively infect or transform beta cells *in vivo.* To guard against the unknown, inducible kill switches could be employed, though these systems face additional challenges.^49^

On a final safety note, should immune evasion strategies not prove sufficient for long-term transplant survival, there is a possibility of utilizing hybrid approaches, pairing edited grafts with milder immunosuppression than what is currently required. While not the perfect solution, this combinatorial approach would likely improve tolerability and safety.

## Clinical progress and challenges for type 1 diabetes

Contingent on successful immune evasion strategies for PSCs, T1D is a leading indication for clinical trials. In pioneering studies, Vertex Pharmaceuticals reported insulin independence and a cure of T1D in a small cohort of patients maintained on immunosuppression and transplanted with unedited hPSC-derived beta cells.^50^ Unfortunately, efforts to protect beta cells from rejection in encapsulation devices were not successful in another trial.^51^

While there is not yet published data in PSCs, Century Therapeutics is pursuing CD300a TASR (trans-antigen signaling receptor) as a potent inhibitor of NK cells, with efficacy demonstrated in HLA-deficient T cells.^33^ Their pipeline includes beta islets derived from HLA-deficient hiPSCs expressing CD300a TASR and an IgG-degrading protease as a treatment for T1D.^46^

Sana Biotechnology has not yet moved hPSC-derived beta islets into clinical trials but instead has begun with cadaveric islets edited using their ‘hypoimmune strategy of ablating HLA-I and -II while overexpressing CD47.^52^ In an n-of-1 pilot study with a noncurative dose of transplanted cells, evidence was provided of graft persistence for at least 12wks.^52^ Given that the impact of overexpressed CD47 on NK cell suppression is controversial, this pilot study may indicate the barrier to allogeneic islet transplantation is lower than had previously been thought, which would be a promising development.

## Conclusion

Our initial expectation was the immune system would find novel ways to reject PSCs even after engineering them to evade known recognition pathways. Yet remarkably, in a variety of *in vivo and ex vivo* xenogeneic and allogeneic settings, in experiments done by independent groups using various strategies, edited PSCs and their progeny evade or tolerize the immune system. The most promising work in this area uses these immune-evasive PSC-derived beta cells to treat T1D. These results are promising enough to warrant some cautious optimism about the potential of PSC therapies to cure T1D without the risks of immunosuppression.

## Data Availability

No new data were generated or analyzed in support of this research.
